# Stigmas, symptom severity and perceived social support predict quality of life for PLHIV in urban Indian context

**DOI:** 10.1186/s12955-016-0556-x

**Published:** 2016-11-03

**Authors:** Helena Garrido-Hernansaiz, Elsa Heylen, Shalini Bharat, Jayashree Ramakrishna, Maria L. Ekstrand

**Affiliations:** 1 Department of Biological and Health Psychology, Psychology Faculty, Universidad Autónoma de Madrid, C/Ivan Pavlov, 6, 28049 Madrid, Spain; 2 Center for AIDS Prevention Studies, Department of Medicine, University of California San Francisco, 550 16th St, 3rd floor, San Francisco, CA 94158 USA; 3 Centre for Health and Social Sciences, School of Health Systems Studies, Tata Institute of Social Sciences, V.N. Purav Marg, Chembur, Mumbai, Maharashtra 400085 India; 4 Department of Mental Health Education, National Institute of Mental Health and Neurosciences, Post Bag 2900, Hosur Road, Bengaluru, 560 029 Karnataka India; 5 St John’s Research Institute, St John’s National Academy of Health Sciences, 100 Feet Rd, Bengaluru, Karnataka 560034 India

**Keywords:** Quality of life, HIV, Stigma, Social support, India

## Abstract

**Background:**

Multiple variables have been studied in relation to health-related quality of life (HRQoL), but research has not integrated the contributions of different variables in a single model that allows to compare them. This study, carried out with people living with HIV/AIDS in India, sought to develop a prediction model considering various predictors previously found to be related to HRQoL, namely sociodemographic factors, HIV symptoms, social support, stigmas and avoidant coping.

**Methods:**

A sample of 961 HIV-positive persons from Bengaluru and Mumbai participated in this cross-sectional study, completing a sociodemographic questionnaire along with HRQoL, HIV symptoms, disclosure expectations, disclosure avoidance, social support and internalized, felt, vicarious and enacted stigma scales. Bivariate associations were obtained (correlations, ANOVAs and *t* tests) and a multiple regression analysis was performed.

**Results:**

Results show that, when all variables are considered together, being married, widowed or deserted, symptom intensity, internalized stigma, disclosure avoidance and enacted stigma contribute negatively to predict HRQoL. On the other hand, being employed, good disclosure expectations and good social support contribute positively to predict HRQoL. Almost half of the variance in HRQoL was explained by this model.

**Conclusions:**

Interventions seeking to increase HRQoL in people living with HIV/AIDS in India would benefit from addressing these aspects.

## Background

Since the introduction of antiretroviral treatments (ART), HIV has been transformed from a fatal disease to a manageable chronic condition that is no longer viewed as a death sentence [1–4]. As a result, People Living with HIV (PLHIV) have longer lifespans, which creates new challenges for health care systems. These are shifting the focus from prolonging PLHIV’s lives to improving their quality of life (QoL), especially the health-related QoL (HRQoL), which is becoming increasingly important as a therapy outcome [5–7] and constitutes a subject of interest for HIV/AIDS researchers on the global level [1].

HRQoL refers to those QoL components that refer to physical and mental health [8], and thus considers not only biological aspects but also psychological and environmental ones [1]. Perception of QoL can directly impact mental health (e.g., depression) and coping skills, and also indirectly by increasing stress, which may in turn affect the immune system and make PLHIV more vulnerable to a variety of conditions. Literature has shown that even when the HIV infection is asymptomatic it has a significant impact on HRQoL [7], which should come as no surprise, as being diagnosed usually leads to a restructuring of PLHIV’s lives. Many uncertainties are faced in relation to health, including HIV-associated co-morbid conditions, side effects of HIV medication, and also AIDS-related complications in those cases where AIDS is developed [7, 9, 10]. Furthermore, a variety of psychosocial challenges are also encountered with regard to personal relationships, financial security, and discrimination, all of which might impact HRQoL [9, 10].

Given this background, the identification of variables predicting HRQoL in PLHIV is key both to better understand the construct and to guide interventions aimed at improving HRQoL [11]. Such identification of predictors can benefit from research that compares the contribution of several potential predictors [5]. Additionally, most of the QoL –and especially HRQoL– field has focused on western settings, mostly in the US, and research in non-western countries such as India with a concentrated HIV epidemic is scarce [5, 12]. Thus our study aims to predict Indian PLHIV’s HRQoL from a set of variables previously found to be related to HRQoL in HIV-positive populations.

### HRQoL measurement in PLHIV

HRQoL focuses on the subjective perception that the individuals have of their physical and mental health, their social relationships and their environment [1]. HRQoL in PLHIV has been assessed via generic instruments or with measures specifically designed to capture the nuances of the HIV condition [13]. 18 HIV-specific and 44 generic HRQoL measures have been found [7], and no instrument seems to be superior to the others. The use of such a wide variety of instruments is a fundamental obstacle to the integration of research findings [5]. The most commonly used HIV-specific scales [7] are The Medical Outcome Study-HIV (MOS-HIV) health survey [14] and WHO Quality Of Life-HIV (WHOQOL-HIV) [15]. The latter is recommended in research aimed to study the effects of HIV (not by comparison with healthy samples) as it considers two important dimensions for PLHIV [1]. It contains 120 items, and its short form, the WHOQOL-HIV-BREF, has 31 [15]. These measures include the dimensions deemed most important by WHO and have been developed and validated in various languages and cultures to ensure cross-culture validity. Their conceptual and operative structure is excellent, as are their psychometric development and reliability [1, 7]. The WHOQOL-HIV-BREF has been previously validated and used in the Indian context [16, 17].

### Variables related to HRQoL in PLHIV

There is a myriad of factors which have been found to influence HRQoL in PLHIV [7]. Among s*ociodemographic variables*, older age and being unemployed are generally related to worse HRQoL, while income and education correlate positively with HRQoL [6, 11, 12, 18]. As for marital status, general literature shows mixed results [11]. In India, it has been found to correlate with some HRQoL facets (negatively with sexual activity and positively with forgiveness) [12], though the study did not specify which marital status categories were used. Having children has been reported as being related to worse HRQoL [11]. Gender differences, for their part, have not been studied much, despite The National Academy of Sciences’ recommendation [19], especially not in non-western countries [12]. Mixed results have been reported in high-income countries [11, 20] and in India – while in one study Indian men showed better HRQoL in the environmental domain and women did on the spirituality/religion and personal beliefs domain [12], other studies have reported no differences [21]. There are two variables related to gender and sexual activity that have not been studied to date in relation to HRQoL. Female sex workers (FSW) and men who have sex with men (MSM) have been historically stigmatized and face many sociocultural barriers such as poverty, exposure to high levels of violence and no access to material or governmental resources [22–24]. Thus, being a FSW or MSM could negatively impact PLHIV’s HRQoL.

As for *medical parameters*, one of the most studied variables has been HIV symptoms [5, 11]. Their prevalence and intensity (stemming from either disease progression or treatment side-effects) have been shown to negatively correlate with PLHIV’s HRQoL [6, 20, 25]. Bing et al. [26] found that even the presence of just one HIV-related symptom significantly reduced HRQoL. ART’s effect on HRQoL, for its part, is complex –it reduces symptoms and improves life expectancy but also generates side effects– and mixed results exist in the literature [11, 27].

Three of the *psychosocial variables* most studied in relation to HRQoL are social support, stigma and coping [5, 11]. Social support, usually diminished in this chronic condition due to the associated stigma [28], has been found to be positively related to HRQoL [11, 18, 29]. HIV stigma is based on the view of PLHIV as people with no moral values [30], and is especially harsh in comparison with stigma related to other diseases [6]. Although experiencing actual rejection and fear of rejection are major stressors of being HIV-positive, and despite the fact that stigma and discrimination have been well documented in Indian populations [24, 31–36], little research has focused on the stigma experienced by PLHIV and its relation to HRQoL in general [20] and particularly in India [30]. Nonetheless, some studies have shown an important negative correlation between the two in China, Africa, Puerto Rico and the US [6, 9, 20, 37].

One type of stigma, internalized stigma –by which PLHIV tend to accept stigmatization from others, feel guilty and justify the discriminatory behavior of others towards them– is quite present in Indian PLHIV [31–33, 38]. It has been consistently negatively correlated with HRQoL [30, 39, 40]. In India, it has been found to be a strong predictor of depression [38]. Other types of stigma, however, such as felt, experienced or vicarious stigma, have received little attention in research [33, 38, 41], as have related constructs such as disclosure expectations or concerns [42].

Regarding coping, an active style (e.g., solving problems, seeking information) has been associated with better HRQoL [11]. A study found that passive coping styles (e.g., disclosure avoidance) did not perform as well as active coping in reducing psychological distress [43], and other research has suggested that disclosure avoidance leads to psychological distress [44]. Although these studies did not consider HRQoL, it is reasonable to postulate that avoidant coping is likely to be negatively related to it.

### Aims of this paper

Our objective is to develop a multiple regression model that considers various predictors previously found to be related to HRQoL, namely some sociodemographic factors, HIV symptoms, social support, stigmas and avoidant coping. It also will consider two additional variables (i.e., stigmatized groups) not previously taken into account (FSW and MSM), and it will allow to study the differential contribution of each variable to HRQoL. Additionally, it will contribute to the knowledge base about HRQoL in PLHIV in India.

## Methods

### Participants

Participants were recruited from two urban areas in India: Bengaluru (formerly Bangalore) and Mumbai. A variety of settings was sampled to ensure diversity, so as to enroll individuals not necessarily in active medical care, to strive for gender balance, and to oversample subpopulations such as MSM and FSW, usually more difficult to reach. These settings included HIV and AIDS nongovernment organizations (NGOs) serving PLHIV and, in Mumbai, also health care settings. Regarding NGOs, study personnel explained the study to local staff and providers and supplied flyers with study information. NGOs staff then referred eligible patients to study personnel. In some cases, the interviewers were able to sit in a private room at the organization and meet with interested individuals. In other cases, referred patients called a study phone line and made appointments with the interviewers. Concerning health care settings, these included HIV units in government hospitals, private for-profit hospitals, not-for-profit government hospitals, and freestanding clinics. After pilot-testing the procedure, everyone who was likely to have at least a 1-h wait was approached by study personnel (patients with less than 1 h wait were generally not interested in participating as they could lose their place in line; moreover, interrupting and continuing the interview after the medical appointment was usually not feasible as the participants had other obligations to fulfill). Eligible individuals had to be at least 18 years of age, self-report diagnosis with HIV/AIDS, and speak English or a local language (Kannada or Tamil in Bengaluru and Hindi or Marathi in Mumbai).

### Instruments

*HRQoL* was measured using the World Health Organization (WHO) Quality of Life (QOL) HIV short version (WHOQOL-HIV BREF) [15], a 31-item measure that assesses the quality of life of PLHIV in six domains: *physical HRQoL*, *psychological HRQoL*, *independence*, *social relations*, *environment*, and s*pirituality/religion/personal beliefs*. All items are answered on a 1 to 5 response scale. Some are negatively worded and need to be reverse-coded so that for all items the higher the score, the higher the HRQoL. This instrument was developed and validated in several countries, including India [16, 17]. For the purpose of this study, only the total score was considered, as other studies have done [6, 20]. We obtained it by adding the scores of the six scales (scores possible range is 24–120). Cronbach’s α in our sample was .92.

*HIV symptom intensity* was assessed via the revised Sign and Symptom Checklist for HIV [45, 46], slightly modified to better fit symptoms reported by Indian patients and then pilot tested. Participants rated the extent to which each of 36 symptoms was a problem to them, ranging from 0 ‘No problem’ to 3 ‘Severe’. All responses were summed to create a symptom intensity variable ranging from 0 to 108.

*HIV related stigma* was assesses in several ways with measures developed and validated in an Indian context [41]. *Felt normative* stigma was the mean of 10 items scored on a 0 ‘No one’ to 3 ‘Most people’ scale, assessing the extent to which participants thought members of their community held stigmatizing opinions of PLHIV (e.g., refuse sharing dishes with PLHIV, avoid visiting PLHIV, think PLHIV are paying for sins/karma,…). Cronbach’s α = .90 in our sample. *Internalized* stigma used the same 10 items to assess how much participants held these beliefs about themselves (0 ‘Not at all to 3 ‘A great deal’). Again, a mean score was created, and Cronbach’s α = .85. The *vicarious* stigma scale contained 10 items assessing how often (0 ‘Never’ to 3 ‘Frequently’) respondents had heard of PLHIV being stigmatized or discriminated in various ways (e.g., ostracized by family or community, hospital worker visibly marking HIV status on medical charts…). A mean score was created over all 10 items; Cronbach’s α = .86. Finally, *enacted* stigma was a tally of the number of ways (out of 10) the respondent had personally experienced HIV related stigma, resulting in an index score between 0 and 10. Cronbach’s α = .72.

*Disclosure expectations* were assessed with the HIV Disclosure Expectations and Repercussions Scale. This measure was created based on Steward et al.’s Study 1 findings about PLHIV’s expectations of what could happen if they disclosed their HIV [41]. We developed a 16-item scale assessing the perceived probability of different outcomes following HIV disclosure (e.g., marriage breakup, physical abuse, rejection or discrimination by peers, family, employers or health professionals, increased support…) and pilot tested it before use in this study. Items are answered on a 4-point scale (1 = Very likely, 4 = Very unlikely). Four items are positively worded and need to be reversed for coding. Scores were derived by averaging responses to the questions, and a higher score indicates better expectations of disclosure outcomes. Cronbach’s α = .87 in this sample.

*Disclosure avoidance* was assessed with the Disclosure Avoidance Scale [41], a 14-item measure assessing the use of strategies (e.g., hiding medications, describing one’s illness as tuberculosis, and seeking care away from one’s local village) to avoid disclosing one’s HIV-positive condition. Items were answered on a 4-point scale ranging from 0 (Never) to 3 (Often). Scores were derived by averaging responses to the questions. Cronbach’s α = .81 in our sample.

*Social support* was calculated as the sum of the dichotomized responses (1 = Yes, 0 = No) to one item asking if the respondents had at least one friend who knew their HIV status and was very supportive, one item about emotional support from family members and another about support from spouse.

*Demographic and medical variables* included ART status, age, marital status, having children, employment, education, place of residence, and gender (male/female/hijra). Hijra is a South Asian term referring to transgender individuals, officially recognized as a third gender by Indian law. Female respondents were further asked if they identified themselves as FSW or if they had sex in exchange for money or goods, and men and hijras were asked if they identified as MSM.

### Procedures

All study procedures were approved by the relevant Indian and U.S. institutions and were conducted in private spaces. Consent was obtained by study personnel before participants completed a structured interview consisting of our various research measures and lasting approximately one hour. Trained interviewers read each question to the participant and recorded the answer on the interview form. It was completed in Kannada, Marathi or Hindi. Since instruments were developed in English, they were translated into the Indian languages, and independently back-translated into English to ensure semantic equivalence [47]. All interviewers underwent training that included basic information about HIV, good interviewing techniques, research ethics, and information about the protocols specific to the study. Interviewers also conducted mock interviews prior to working in the field and were supervised by study investigators. A small monetary token was given to participants.

### Data analysis

Descriptive univariate statistics consisted of frequencies and percentages for categorical variables and means and standard deviations (*SD*) for scale and index variables, which were treated as continuous variables. Bivariate associations between HRQoL and other continuous variables (stigma types, disclosure expectations, disclosure avoidance, social support, and symptom intensity) were assessed via Pearson’s correlation coefficients. Differences in mean level of HRQoL between categories of dichotomous variables (city of data collection, being a FSW or MSM, being employed, having children, being on ART) were assessed via *t*-test. For multiple-category variables (gender group, education, marital status, religion), one-way ANOVA was used, with post-hoc Bonferroni-corrected between group comparisons in case of a significant overall F-value.

Variables that were associated with HRQoL at *p* < .05 in the analyses were subsequently entered into a multiple linear regression model. No evidence was found of multicollinearity between the independent variables (all VIF and tolerance values were, respectively, < 2.5 and > .40) [48]. Residuals were examined for non-normality, heteroscedasticity and influential outliers (via Cook’s distance D), and none seemed problematic. All significance levels reported are two-sided. Analyses were performed in SPSS v22.

## Results

### Sample descriptive analyses

Table 1 shows the sample characteristics and the mean in HRQoL for each group. Approximately half the participants were recruited in Bengaluru and half in Mumbai. The mean age was 33.12 (*SD* = 7.16) and the mean monthly income was Rs 4548.18 (*SD* = 6674.39). Over half the participants were female, and more than a third of the females were FSW, while only around 16 % of males and hijras identified as MSM. Most of the sample was Hindu, married, with children and employed. A quarter of the participants could neither read nor write, and about half had received 5–10 years of education. Slightly over half of the participants were taking ART. The mean HRQoL (*M* = 83.78) was similar to that found in other studies in India [12].

**Table 1:** Demographic and medical characteristics of the sample

	Mean	*SD*	Range
Age (years)	33.12	7.16	18–68
Household monthly income (Rupees)*	4548.18	6674.39	0–150,000
	*N*	%	HRQoL M (*SD*)
Gender			
Male	396	41.2	84.82 (17.29)
Female	534	55.6	82.94 (17.01)
Hijra	31	3.2	84.50 (20.39)
Female			
Sex worker	208	39.0	82.55 (16.27)
Not sex worker	326	61.0	83.20 (17.49)
Male/Hijra			
Have sex with men	68	15.9	83.71 (16.92)
Not have sex with men	359	84.1	86.24 (15.03)
Site*			
Bangalore	511	53.2	87.06 (15.29)
Mumbai	450	46.8	79.29 (18.73)
Religion			
Hindu	777	80.9	84.46 (16.83)
Muslim	98	10.2	79.10 (19.07)
Christian	51	5.3	83.61 (18.53)
Buddhist	30	3.1	78.49 (17.27)
Other	5	.5	96.70 (16.94)
Marital status*			
Never married/single^a^	154	16.0	88.28 (17.45)
Currently married^b^	430	44.7	83.27 (17.67)
Divorced/separated^a,b^	57	5.9	84.36 (16.60)
Widow/widower^b^	255	26.5	82.80 (16.32)
Deserted^b^	64	6.7	80.52 (16.92)
Children*			
No	256	26.6	87.03 (16.69)
Yes	705	73.4	82.62 (17.31)
Educational level*			
None^a^	245	25.5	79.57 (16.80)
7 years or less^a,b^	249	25.9	82.22 (17.93)
8–10 years^b,c^	331	34.4	86.35 (16.82)
More than 10 years^c^	134	13.9	87.74 (16.00)
Currently employed*			
Yes	743	77.3	85.05 (16.82)
No	218	22.7	79.35 (18.02)
Taking ART			
Yes	528	54.9	83.12 (17.49)
No	433	45.1	84.59 (16.94)

The sample (*N* = 961) was collected in Bangalore and Mumbai, India, between 2007 and 2009. The asterisk (*) indicates variables with a significant correlation with HRQoL or with significant HRQoL mean differences among categories. For variables with more than two categories, those with a different superscript letter show a significant difference in HRQoL mean

*HRQoL* health-related quality of life, *M* mean, *SD* standard deviation, *N* number of participants, % percentage of participants, *ART* antiretroviral treatment

### Bivariate associations

Participants from Bengaluru had higher HRQoL (*M* = 87.06) than those from Mumbai (*M* = 79.29; *t*[697.50] = 6.55, *p* < .001), as did participants who were employed (*M* = 85.05) compared to their unemployed counterparts (*M* = 79.35; *t*[877] = −4.11, *p* < .001), and childless participants (*M* = 87.03) in comparison to those with children (*M* = 82.62; *t*[877] = 3.37, *p* = .001). No differences in HRQoL were found between FSW and the rest of the females (*t*[477] = .41, *p* = .683), between MSM and the rest of the males/hijras (*t*[412] = 1.22, *p* = .223), or between those on ART and those who were not (*t*[877] = 1.26, *p* = .208).

Differences appeared regarding marital status (*F*[4, 878] = 3.18, *p* = .013), and post-hoc Bonferroni test showed that single participants had higher HRQoL (*M* = 88.28) than currently married (*M* = 83.27), widowed (*M* = 82.80), and deserted participants (*M* = 80.52). A difference was also found by educational level (*F*[4, 876] = 7.30, *p* < .001). Post-hoc Bonferroni test showed that those participants with eight to 10 years of education (*M* = 86.35) and those with more than 10 (*M* = 87.74) had better HRQoL than those who had received none (*M* = 79.57). Likewise, those participants with more than 10 years of education (*M* = 87.74) had better HRQoL than those with seven or less (*M* = 82.22). Although he *F* statistic was significant for religion (*F*[4, 878] = 3.19, *p* = .013), post-hoc Bonferroni did not show differences (lowest *p* = .069). No gender differences emerged, either (*F*[2, 878] = 1.26, *p* = .285).

HRQoL’s correlations with age and income were, respectively, *r* = −.03 (*p* = .46) and *r* = .12 (*p* = .001). Correlations among HRQoL, the different stigma types, disclosure expectations, disclosure avoidance, social support, and symptom intensity are presented in Table 2. Most correlations were significant, although some of them are low, especially among the different stigma types. Correlations between HRQoL and other constructs were all significant, with absolute values ranging from .16 to .46. The strongest correlations were with disclosure expectations (*r* = .46), symptom intensity (*r* = −.42), internalized stigma, and disclosure avoidance (both *r* = −.37; all *p* < .001).

**Table 2:** Correlations among HRQoL, stigma types, disclosure avoidance, disclosure expectations, symptom severity and social support

	1	2	3	4	5	6	7	8
1. HRQoL								
2. Internalized stigma	−.37***							
3. Felt stigma	−.28***	.21***						
4. Vicarious stigma	−.16***	−.02	.26***					
5. Enacted stigma	−.25***	−.01	.17***	.45***				
6. Social support	.35***	−.09**	−.21***	−.10**	−.09**			
7. Symptom severity	−.42***	.22***	.15***	.10**	.17***	−.08*		
8. Disclosure avoidance	−.37***	.24***	.31***	.21***	.17***	−.12**	.19***	
9. Disclosure expectations	.46***	−.17***	−.38***	−.26***	−.26***	.39***	−.24***	−.36***

Table shows Pearson’s correlations among measures

*HRQoL* health-related quality of life

***p < .001

**p < .01

*p < .05

### Multiple linear regression analysis

Based on their *p*-value < .05 in the bivariate analyses, the following variables were entered into a multiple linear regression model with HRQoL as the dependent variable: marital status (reference = single), site of data collection, employment status, household income, educational level, having children, symptom severity, social support, felt stigma, internalized stigma, vicarious stigma, enacted stigma, disclosure expectations and disclosure avoidance. A significant regression equation was found (*F*[17,837] = 46.13, *p* < .001; adjusted *R* ^*2*^ = .48). There were several variables that were not significant predictors: having children (*t* = −.22, *p* = .824), income (*t* = 1.29, *p* = .199), educational level (*t* = 1.31, *p* = .191), felt stigma (*t* = −1.95, *p* = .052) and vicarious stigma (*t* = 1.32, *p* = .186). The regression model was calculated again without these variables (*F*[12,859] = 64.08, *p* < .001, adjusted *R* ^*2*^ = .47) and the detailed results of this final model are shown in Table 3. The variables contributing the most to the prediction were symptom intensity (*β* = −.26), internalized stigma (*β* = −.21), disclosure expectations and social support (both *β* = .20).

**Table 3:** Multiple linear regression of health-related quality of life

	Unstandardized coefficients		Standardized coefficients
	B	S.E.	*β*
Marital status (ref.: single/never married)			
Married	−6.23	1.30	−.18***
Divorced/separated	−.62	2.06	−.01
Widow/widower	−4.79	1.38	−.12**
Deserted	−4.35	1.98	−.07*
Site (ref.: Bengaluru)	−4.57	.90	−.13***
Employed	3.46	1.03	.08**
Symptom intensity	−.36	.04	−.26***
Social support	3.51	.50	.20***
Internalized stigma	−5.03	.65	−.21***
Enacted stigma	−.87	.23	−.10***
Disclosure avoidance	3.66	.77	−.13***
Disclosure expectations	4.75	.71	.20***

Only the significant coefficients are included in this final model. *N* = 860

*S.E.* standard error

***p < .001

**p < .01

*p < .05

## Discussion

This study identified variables predicting HRQoL in PLHIV in India and integrated their contribution in a single regression model, which explained almost half of the variance in HRQoL. The variables marital status (being married, deserted or a widow/widower compared to being single), symptom intensity, internalized stigma, disclosure avoidance and enacted stigma contributed negatively to predict HRQoL. On the other hand, living in Bengaluru, being employed and having good disclosure expectations and social support contributed positively to predict HRQoL.

HRQoL has been previously associated with social stigma variables and intensity of symptoms in a correlational study with a sample of 55 African American men [6], as well as with social support in a correlational study with a sample of 120 PLHIV in Beijing, China [18], and in Degroote et al.’s recent review of the literature on HRQoL determinants [11]. With regard to social stigma variables, our study provides additional insight in relation to the differential weight of stigma types: internalized stigma seemed to be twice as important to HRQoL prediction as enacted stigma, while vicarious and felt stigmas did not contribute significantly to prediction in spite of showing a bivariate association to HRQoL. Internalized stigma has been previously reported as more important than other stigma types in South India [30]. The negative contribution of disclosure avoidance as a form of avoidant coping is also coherent with previous findings, such as a study with PLHIV in South Africa [44] and a literature review on coping and QoL in PLHIV [43]. The positive contribution of being employed has been well established in literature, as Degroote et al.’s review demonstrates [11]. The relation we found between good disclosure expectations and better HRQoL, is consistent with a study which found that more disclosure concerns were associated with poorer HIV QoL [42]. Lastly, regarding marital status, being single has been previously found to be associated with better HRQoL in women, whereas being married was associated with a decline [49]. This is congruous with our findings, which also show that being a widow/widower or deserted is related to worse HRQoL.

There were some variables that showed bivariate associations with HRQoL but did not significantly predict HRQoL when all were considered together. In addition to the abovementioned vicarious and felt stigma, this was the case for having children, education and income. Regarding education, this finding is coherent with a study among African American men which also found a significant bivariate correlation to HRQoL but no significant weight in regression analyses [6]. Having children has also been previously associated to worse HRQoL, particularly to physical health according to Degroote et al.’s HRQoL literature review [11]. It seems that having children is a source of support but can also be a source of stress and concern, and its effect on HRQoL might depend on other aspects, such as health status or infection stage. As for income, the same review warns that the information an income variable provides is limited, as other related aspects could inform better of the financial status of the person (e.g., expenditures, family composition, financial insecurity and worries). Moreover, it might be that another variable, employment status, captures better the economic aspects, and it also may affect HRQoL in other ways, as it provides structure to daily life, a social support network and role identity [11].

Some of the variables included in our study did not show even a bivariate association to HRQoL. The lack of relation between gender and HRQoL is consistent with some studies conducted in north India [21] and with large (*N* = 726) international samples [20], although other studies have revealed gender differences as stated in Degroote’s review and in a study in south India [11, 12]. The same happens with ART status [11], and so our results only add to those mixed findings in previous literature. Regarding age, the lack of association to HRQoL is coherent with previous research in African American men [6] and findings about certain HRQoL dimensions [11]. Lastly, being a FSW or MSM was not related to HRQoL, which was an unexpected result warranting further research, as no studies previously took an interest in this aspect.

### Limitations

Although our work has clarified to some extent the contribution to HRQoL prediction in India, some limitations need to be taken into account. The fact that data collection was carried out in Mumbai and Bengaluru limits the generalizability of the results to rural Indian populations. The cross-sectional design prevented the exploration of the directionality of the associations, so a causal model remains to be tested. Lastly, the variables included in our research design were limited, as other aspects could contribute to HRQoL prediction, such as satisfaction with care, adherence, substance use, viral load, CD4 count, stage of infection, time since diagnosis or comorbidity [11, 27]. Further research should consider and overcome these limitations to advance knowledge on HRQoL.

### Recommendations

Our work has important implications despite its limitations. Interventions aimed at improving HRQoL of PLHIV in urban India should focus, in the first place, on reducing HIV symptoms. This could be achieved by improving early diagnosis and access to medication, and by reducing ART side-effects. Interventions also should implement mechanisms to improve disclosure expectations of PLHIV and foster their disclosure to significant ones (e.g., by training them in disclosing techniques and providing HIV support programs for family and friends). The reduction of enacted stigma and promotion of a good social support network are also key aspects for intervention that could be tackled by means of HIV support programs for PLHIV and for family and friends. Efforts could also be made to reduce internalized stigma of PLHIV with specific interventions that challenge their hostile attitudes toward the infection. Lastly, programs aimed at improving HRQoL may want to encourage employment PLHIV, which could be accomplished by providing them with job-specific education or training and by teaching them job-finding skills (e.g., open position search skills, interview skills, etc.).

## Conclusions

A number of variables (marital status, employment status, place of residence, symptom intensity, internalized stigma, disclosure avoidance, enacted stigma, disclosure expectations, and social support) are related to HRQoL even when considered together. More research is needed to clarify the effect of some of them, as literature shows mixed results, but the effect of others is consistent through research and should be addressed in comprehensive psychological interventions to increase HRQoL in PLHIV in urban India.

## Abbreviations

ART: Antiretroviral treatments
FSW: Female sex workers
HRQoL: Health-related quality of life
MOS-HIV: Medical outcome study-HIV
MSM: Men who have sex with men
NGO: Nongovernment organization
PLHIV: People living with HIV
QoL: Quality of life
SD: Standard deviation
WHOQOL-HIV: World Health Organization quality of life-HIV
WHOQOL-HIV-BREF: World Health Organization quality of life-HIV short form
